# Experimental Study of High Performance Synchronous Grouting Materials Prepared with Clay

**DOI:** 10.3390/ma14061362

**Published:** 2021-03-11

**Authors:** Ying Cui, Zhongsheng Tan

**Affiliations:** 1 School of Civil Engineering, Beijing Jiaotong University, Beijing 100044, China; cuiying@bjtu.edu.cn; 2 Key Laboratory for Urban Underground Engineering of Ministry of Education, Beijing Jiaotong University, Beijing 100044, China

**Keywords:** synchronous grouting, grouting material, clay, epoxy resin

## Abstract

Shield construction discharges a large amount of soil and muck. The utilization of discharged soil of shield always has high energy consumption and a low utilization rate. Meanwhile, synchronous grouting is a key process for shield tunneling. The current studies show that the synchronous grouting materials applied now generally have the problem of mismatching among filling property, fluidity, and consolidation strength. In order to study the feasibility of using the excavated soil produced by shield construction in clay stratum as synchronous grouting material, high performance synchronous grouting material was studied by taking red clay as an example, modified by epoxy resin. The fluidity, stability, and strength were measured to evaluate performance of the grout. Material test results show that the addition of waterborne epoxy resin decreases density, improves the stability, the rate of stone, and the toughness of the grouting concretion. Finally, X-ray diffraction (XRD) and Scanning Electron Microscopy (SEM) were measured to analyze the cementitious mechanism of the grout, test results demonstrated that cement hydration and curing reaction of epoxy resin happened in the grout, the formed polymer film filled the voids in the mixture and effectively bound cement hydration gel and clay particles together.

## 1. Introduction

**Figure 1:**
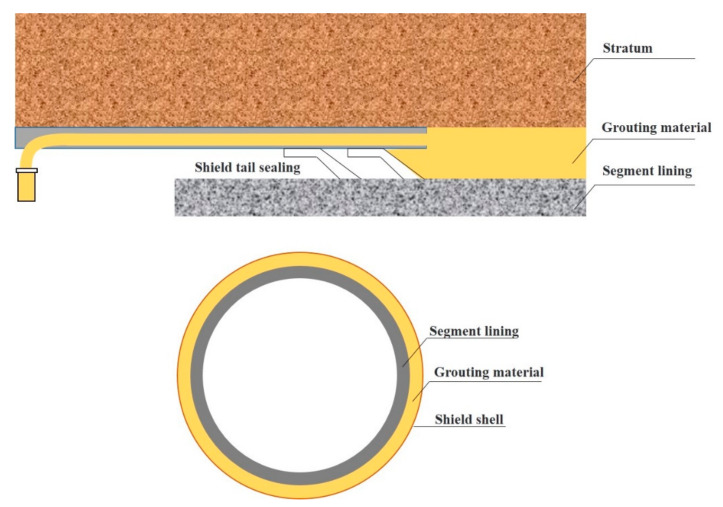
Synchronous grouting in shield tunneling.

Shield tunneling technology is the main method for urban underground and underwater tunnel construction. Synchronous grouting is a necessary and key process for shield tunneling. Grouting material is pressed into the void at the end of the shield to prevent segment ruptured due to uneven force [1,2] (Figure 1). Various types of synchronous grouting materials have been studied to meet the needs of different construction [3]. Inert grout is the grout without cement and other gel materials, the early and late strength of the grout are low, consolidation time is long, and volume shrinkage occurs during consolidation. However, inert grout has an obvious advantage of good liquidity, which can solve the problem of pipeline blockage in synchronous grouting [4]. Zhu et al. [5] proposed an optimal preparation method for inert grout with improvement of dynamic and static properties, and carried out a mixture ratio test. Xiao et al. [6] analyzed the influence of thickener and sodium hydroxide on the performance of inert grout, and obtained the optimized ratio. The single-fluid grout has the characteristics of long setting time and quite simple grouting technology, so it is widely used in many practical projects [7]. Wang et al. [8] prepared backfill grouting material with organic-inorganic composite technology and applied it in the Wuhan Yangtze River Tunnel. Peng et al. [9] added styrene-acrylic emulsion, pure acrylic emulsion, and emulsified asphalt in the high content fly ash synchronous grouting material, the crack resistance and water resistance of material were improved. In recent years, the two-component grout has begun to be used in the shield tunnel construction, especially in water-rich strata, due to its short gel time, high early strength, and good stability [7]. Su et al. [10] conducted a grouting simulation experiment, and discussed the influence of each component on the fluidity, stability, strength, and other properties of the grout. The two-component grout has been commonly used in Japan, Italy, New Zealand, Bulgaria, and Singapore [4]. However, the two-component grout blocks the pipe easily, and the grouting process is complicated. At present, the grouting materials studied and applied generally have the problem of mismatching among filling property, fluidity, and consolidation strength [11]. Therefore, it is important to develop a new green synchronous grouting material with good stability, high hardening strength, and good fluidity using pollution-free raw materials.

Meanwhile, about 50,000 m^3^ waste soil is produced during shield tunneling construction per km. A large amount of discharged soil and muck needs to be transported, accumulated, and absorbed, causing great environmental and economic problems [12]. Due to the different geology and complex composition of the discharged soil, systematic utilization is difficult. Several studies have been reported for utilization of discharged soil of shield, such as preparing ceramsite and brick. Li et al. [13] prepared a kind of ceramsite for phosphorus removal in water by using shield muck and modified it with magnesium oxide at low temperature. Zhang et al. [14] used discharged soil of shield and fly ash to prepare ceramsite by sintering. Grohs [15] found that after a simple treatment, the discharged soil of shield could replace a portion of the construction raw materials. Jiang et al. [16] pointed out that the physical properties and chemical compositions of the shield discharged soil were suitable for sintering brick. However, most of the utilization methods of shield muck are applied with high energy consumption and low utilization rate, which makes it difficult for large-scale application [17]. If discharged soil of shield can be used as synchronous grouting, the cost of soil treatment and environmental pollution would be greatly reduced. Liang et al. [18] prepared backfill grouting material by shield muck of fine silty sand layer. Zhong et al. [19] studied the reuse of fine sand discharged from shield excavation for grouting behind the lining. Zhou et al. [20] explored in-situ recycle of excavated sandy soil in the Nanjing Yangtze River tunnel project as the back-fill grout instead of purchased sand. Zhang et al. [21] used waste mud of slurry shield and fine sand as synchronous grouting materials. Xu et al. [22] proposed that shield muck could replace bentonite and river sand. By adjusting and optimizing the mix ratio through orthogonal test method, the mix ratio of series of synchronous grouting materials suitable for different conditions was given. Yang et al. [23] used waste mud to prepare backfill grouting mortar. Zhang et al. [12] prepared synchronous grouting material with discharged soil from the Zhengzhou metro line 3 project in China. The discharged soil could be used by mixing with clayey or sandy content in a proper mass ratio after treatment. Zhou et al. [24] improved the inert slurry by using discharged soil of shield. However, most of the existing studies focus on the reuse of silty sand shield muck and lack of research on the reuse of clay shield muck as grouting material.

A kind of high performance synchronous grouting material was prepared by using red clay in this research, and its mechanical properties and microstructure were studied, for providing a theoretical basis for the application of excavated soil produced by shield construction in clay stratum as synchronous grouting material.

## 2. Materials and Methods

### 2.1. Materials

In this study, the ordinary Portland cement produced by Hebei Yanxin Building Materials Co., LTD. (Langfang, China) was used in the experiment. The chemical composition of the cement was analyzed according to the Chinese Standard GB/T 176-2017 [25] by using Panalytical Axios X-ray fluorescence equipment, the result is presented in Table 1. Red clay produced by Hebei Zhanghan Mine (Cangzhou, China) was selected as raw materials for this research. The chemical composition of the clay was measured by using Panalytical Axios X-ray fluorescence equipment. The mineral components were measured according to Chinese Standard SY/T 5163-2010 [26] by using X-ray diffractometer, samples under three different treatment conditions were tested, and the K-value method was used for quantitative analysis. The test results are listed in Table 2 and Table 3. Water-soluble epoxy resin (ER) is one of the principal resins used for polymer modified concretes and mortars, for its great advantage of increasing compressive, flexural strengths, and workability, reducing segregation and shrinkage [27]. Bisphenol-A water-based resin used in the mixture was produced by Baling Petrochemical company (Yueyang, China), composed of water-based resin and water-based curing hardener. The optimum mixture ratio by weight of epoxy resin (A) and hardener (B) was A:B = 1:1. The epoxy equivalent was 190–210 g/eq, viscosity was 500–1500 mpa·s. Additionally, UNF-5 naphthalene series superplasticizer produced by Beijing Muhu Admixture Co., Ltd. (Beijing, China) was used, water reduction rate ≥12%.

### 2.2. Mixing and Testing Procedures

In order to achieve good grouting effect behind the lining, the grouting material should have: Low drainage rate, high rate of stone, good fluidity, fully flow to the filling part before consolidation, a certain early strength, and eco-friendly [28]. In this paper, experiments were carried out to study the effect of each component on performance of the grout. The dosage scopes of the investigated materials were determined according to previous experience in grouting engineering and several primary experiments. Cement, clay, and superplasticizer were mixed in proportion (as in Table 4) with a water to solid ratio of 0.5, water was added to stir uniformly, then epoxy resin emulsion (Baling Petrochemical company, Yueyang, China) and curing agent (Baling Petrochemical company, Yueyang, China) were added according to the proportion (listed in Table 4), mechanical stir 120s to uniform slurry, and loaded into the test mold. It was formed by 40 mm× 40 mm × 160 mm triple steel mold, which is demodulated 1d later, cured to the corresponding age in the standard curing chamber with temperature (20 ± 2) ℃ and relative humidity ≥95%.

The density of the grout with different proportion ratios was determined by cement densimeter (Cangzhou Shouke Instrument Equipment, Cangzhou, China) according to the Chinese standard GB/T 19139-2003 [29]. Setting time of the grout was conducted by the Vicat apparatus (Cangzhou Shouke Instrument Equipment, Cangzhou, China). The grout was poured into a test mold and put under the test needle and the needle made contact with the surface of the sample, suddenly relaxed the test needle, when the test needle sank into 41 mm away from the bottom of the mold, the time was recorded, as the cement reached the initial setting state. The final setting time was recorded when the test needle sank in 0.5 mm of the sample. The fluidity was measured by conical mold testing according to the Chinese Standard GB/T 8077-2000 [30]. Fresh grout was filled into the conical mold (height 6 cm, top diameter 3.6 cm, and bottom diameter 6 cm), then the conical mold was lifted in the vertical direction, while at the same time the stopwatch was opened for timing, and the grout flowed freely on the glass plate (400 × 400 ×5 mm^3^). After 30 s, the maximum diameters of the grout at two different directions perpendicular to each other were measured with a ruler, which took the average value as the fluidity. The bleeding rate and stone rate of the grout effect the grouting result directly. The prepared grout was poured into the measuring cylinder (capacity: 250 mL) to about 200 mL immediately after being evenly stirred, we recorded the amount of water after 2 h, and then calculated the bleeding rate of the grout. The test method of stone rate was the same as that of the bleeding rate test. The curing time was extended to 24 h on the basis of bleeding rate measurement. The stone rate of the grout can be obtained by comparing the volume of the stone body with the original volume of the grout. Compressive strength and flexural strength at 3 days, 7 days, and 28 days were measured according to Chinese Standard GB/T 17671-1999 [31], cubic specimens (40 × 40 × 160 mm) were tested by using a WAW-600D microcomputer control electro-hydraulic servo universal testing machine (Shanghai Tuofeng Instrument Technology, Shanghai, China) after curing for 3, 7, and 28 days under the standard curing conditions (humidity is more than 95%, temperature is 20 ± 1 ℃). The compressive strength was measured by fracture blocks of flexural specimens and the compression speed during the test was uniformly controlled at 0.5 mm/min. When the specimen failed, the peak value of force was recorded. Each group of samples was tested three times, and the average value was calculated.

The microstructural characters and chemical reactions in the grout were conducted through Scanning Electron Microscopy (SEM) and X-ray diffraction analysis (XRD). All test samples were taken from the sample blocks damaged by strength test, soaked in anhydrous ethanol to stop hydration, dried at 60 ℃ for 24 h before test. A Bruker D8 Advance X-ray diffractometer (Bruker, Karlsruhe, Germany) was used. Anode Material was Cu, voltage was 40 kV, current was 40 mA, and the samples were ground into powder before the test. For SEM test, the sample was coated with film on a double-sided conductive metal table, and then the microscopic morphology were observed by JSM-6390 A scanning electron microscope (JEOL, Tokyo, Japan) with different magnifications.

## 3. Results and Discussion

### 3.1. Workability

**Figure 2:**
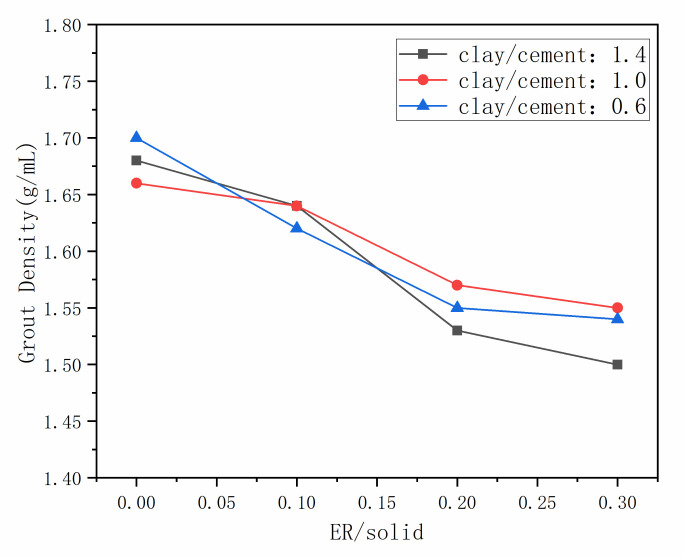
The density of grout.

**Figure 3:**
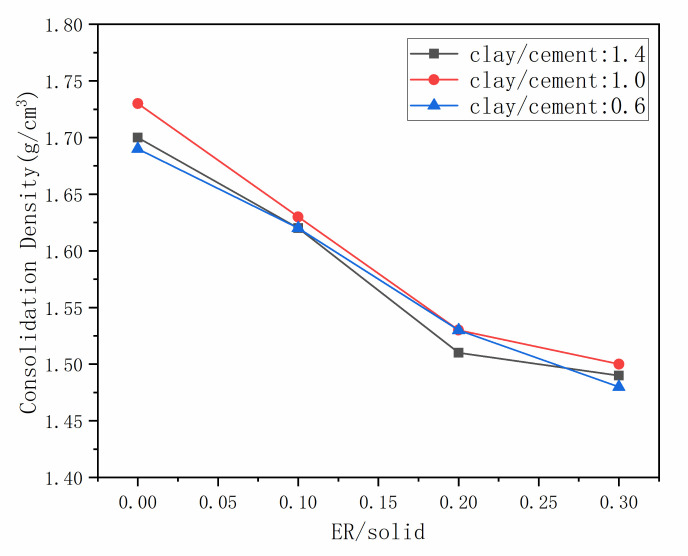
The density of grout consolidation.

The density of the grout and consolidation with different proportion ratios were measured, with the results shown in Figure 2 and Figure 3. As shown in Figure 2, the density of the grout decreases with the increment of epoxy resin. The fresh grout is a complex multiphase system with solid-liquid-gas. The solidified resin, hydrated products, and clay particles influence and restrict each other in the composite system, the surface activity of resin particles has great influence on the process of grout consolidation. After the addition of resin, the viscosity of fluid phase in the grout was increased, and air was introduced during stirring process, which reduced the density of grout [32]. Meanwhile, the density of clay and cement is similar, the density of resin is less than cement clay slurry. In the mixed grout, with the increase of low-density components, the overall density of the grout decreased. From Figure 3, the density of the consolidation also decreased with the increment of epoxy resin, and it appears not much different from the grout density. This is because during the grout consolidation process, the water in the mixed system participates in the hydration reaction of cement and the curing reaction of resin. Resin particles fill and seal the pores of the hydration products and clay particles, which can prevent water evaporation [32].

Table 5 shows the setting time of the grout with different proportion ratios. As demonstrated in Table 5, the setting time of the grout gradually increased with the increase of epoxy resin and clay dosage. In the hydration process of cement, due to the effect of hydrophilic groups such as hydroxyl and ester group, the polymer adsorbed on the solid-liquid interface enveloped the cement hydration particles, impeded the hydration of cement. Secondly, polymer adsorbed on the cement particle makes the hydration layer thickening, condensation of cement particles is prevented, and the hydration of the cement was delayed. Similar results were also reported by Su et al. [33] and Anagnostopoulos [34]. In addition, with the increase of clay content, the setting time increase, this is because the addition of clay particles dispersed cement particles, the cement hydration reaction is impeded, also the reaction between clay particles and hydration products is relatively slow, which affects the setting time of the grout [35].

**Figure 4:**
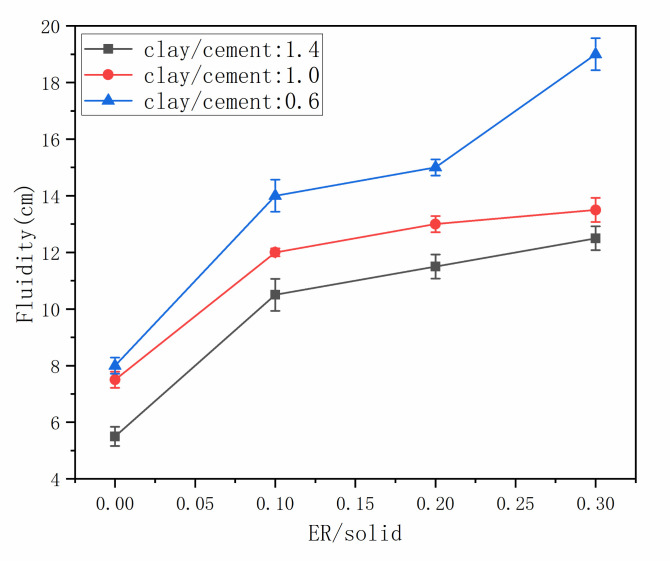
The fluidity of grout.

**Figure 5:**
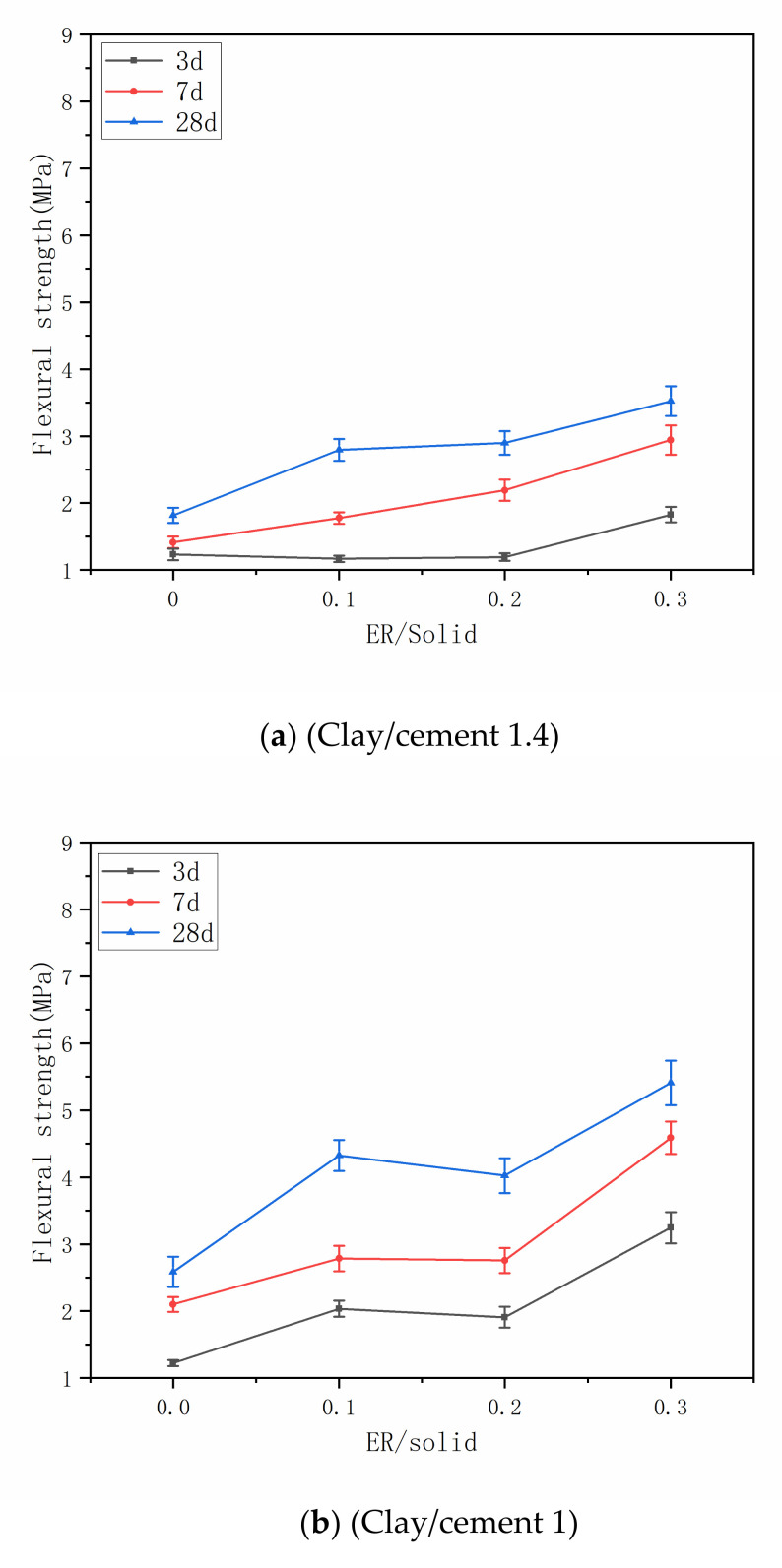

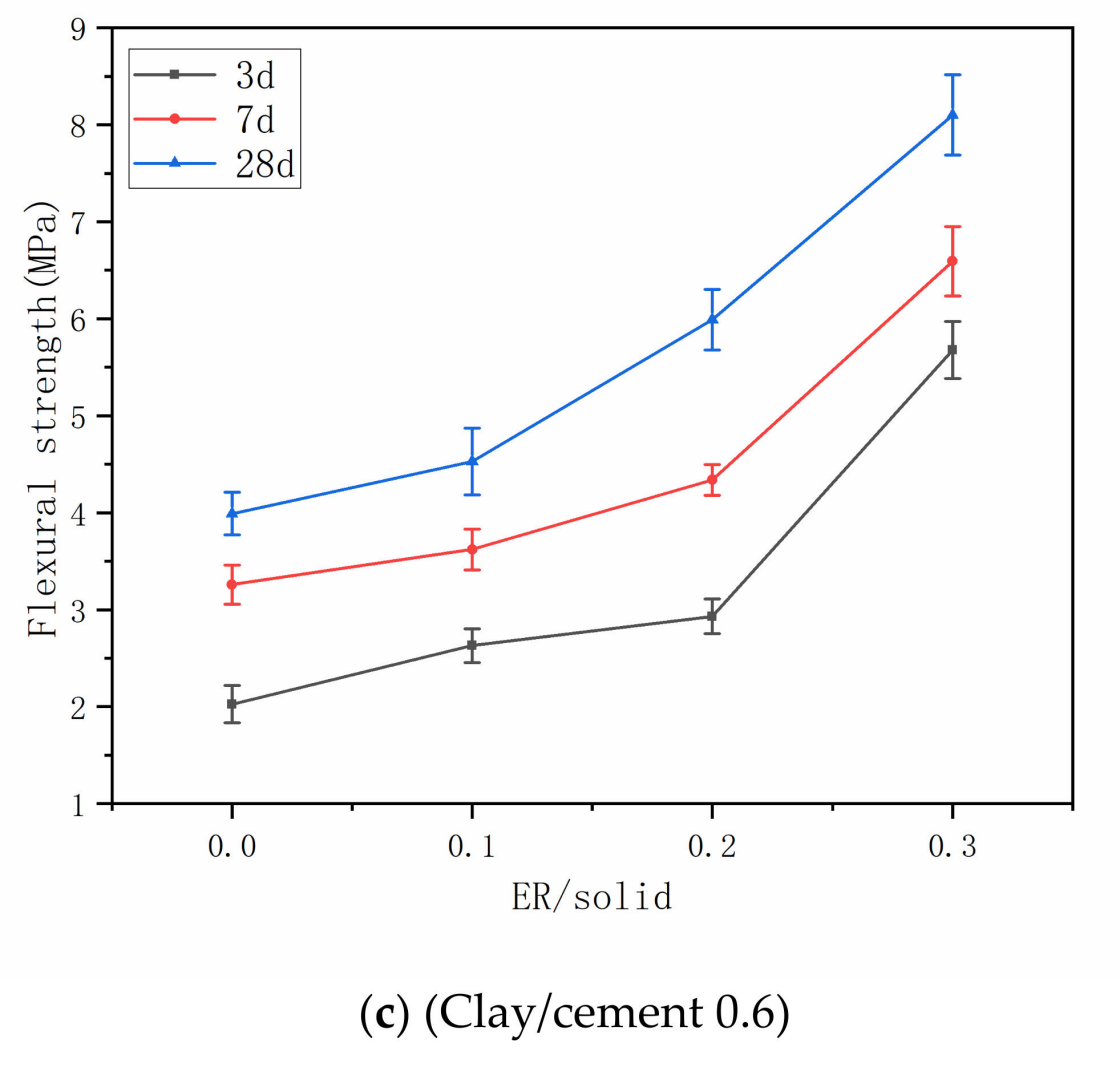
Flexural strength of the grouts with different ER/solid and clay/cement. (**a**) Clay/cement 1.4; (**b**) Clay/cement 1; (**c**) Clay/cement 0.6.

The fluidity of grouting material determines its engineering practicability. The fluidity of the grout with different epoxy resin and clay dosage were measured, with the results shown in Figure 4. It can be observed that the fluidity increased with the increment of epoxy resin. This is because when epoxy resin particles are adsorbed on the surface of cement particles and clay particles, the surface of cement particles has the same charge, the repulsive force between particles increases, and the dispersion of cement particles and clay particles is enhanced, and the flocculent structure in the original cement clay slurry is separated. Such results agree with the results presented by previous studies [36,37,38]. In addition, Figure 5 shows that the fluidity decreased with the increase in clay dosage. This can be attributed to the great water absorption of clay. Clay particles have large surface area and charged layer structure, which can attract water molecules [39,40].

### 3.2. Stability

For grouting engineering, the bleeding rate of grout is an important factor to evaluate the stability of the grout. When the grout with high bleeding rate is flowing, the viscosity of the grout surface decreases due to precipitated water. With the settling of particles in the grout, the viscosity of the grout at the lower part increases, leading to the decrease of the effective diffusion distance of the grout. Additionally, the grout with high bleeding rate will shrink during the consolidating process, and the strength of the consolidation vary from top to bottom, which is not conducive for grouting. The bleeding rate and stone rate of the grout with different proportion ratios were measured with the results shown in Table 6. It is observed that the addition of epoxy resin has little effect on the bleed rate and the stone rate of the grout. However, when the proportion of clay/cement in the grout was 0.6, the bleeding rate was higher than that in the grout with clay/cement proportion of 1.4 and 1, also the stone rate was lower. The results indicated that the clay decreased the bleeding rate and increased the stone rate of the grout. Several studies demonstrated similar trends of bleeding rate [41]. In the grout, a large number of active ions were produced in cement hydration reaction. The exchanges among Na^+^, K^+^, and Ca^2+^ would be accelerated by these active ions, and the bonding force between particles was strengthened. As a result, the bleeding rate decreased, and the stone rate increased accordingly [42]. Due to such ability of clay, when the water-solid ratio is 0.5, the bleeding rate of the grout is below 1%, and the stone rate of the grout reaches more than 97%, which can fully meet the requirements of synchronous grouting.

### 3.3. Mechanical Properties

Figure 5 shows influence of epoxy resin on the flexural strength. It can be seen that with the increase of curing age, the flexural strength of each grout mix increases, and the addition of epoxy resin increases the growth rate of flexural strength. Additionally, epoxy resin plays a positive role on the flexural strength, which agrees with the test results of previous studies [43]. After epoxy resin emulsion and curing agent mixed with cement clay slurry, the emulsion particles dispersed in water with the curing agent molecules. As the water evaporated, epoxy resin emulsion particles make contact with each other and form a stack structure. At the same time, curing agent molecules spread to epoxy resin particle interface and begin their internal curing reaction, small epoxy resin molecule polymerized into macromolecule. The whole process is carried out at the same time as cement hydration, the polymer is gradually distributed in the matrix, and the polymer film is formed gradually. These films have a certain elasticity, which reduces the brittleness of cement-based materials. Besides, some active groups in the water-based epoxy resin may react with the hydration products to form a special bridge bond and cross with the hydration products, which can inhibit the development of cracks and improve the flexural strength of the stone body [32].

**Figure 6:**
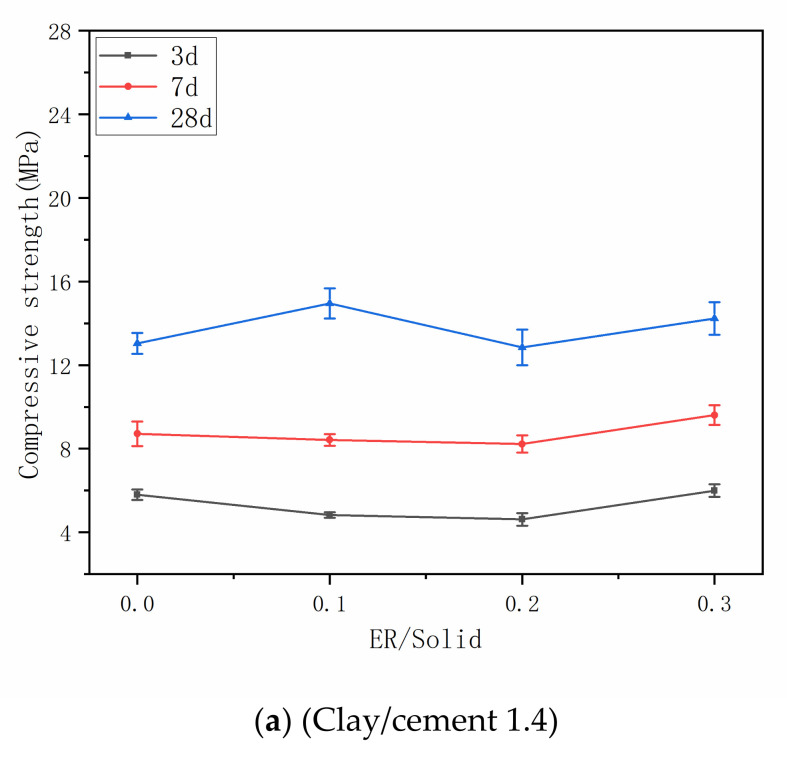

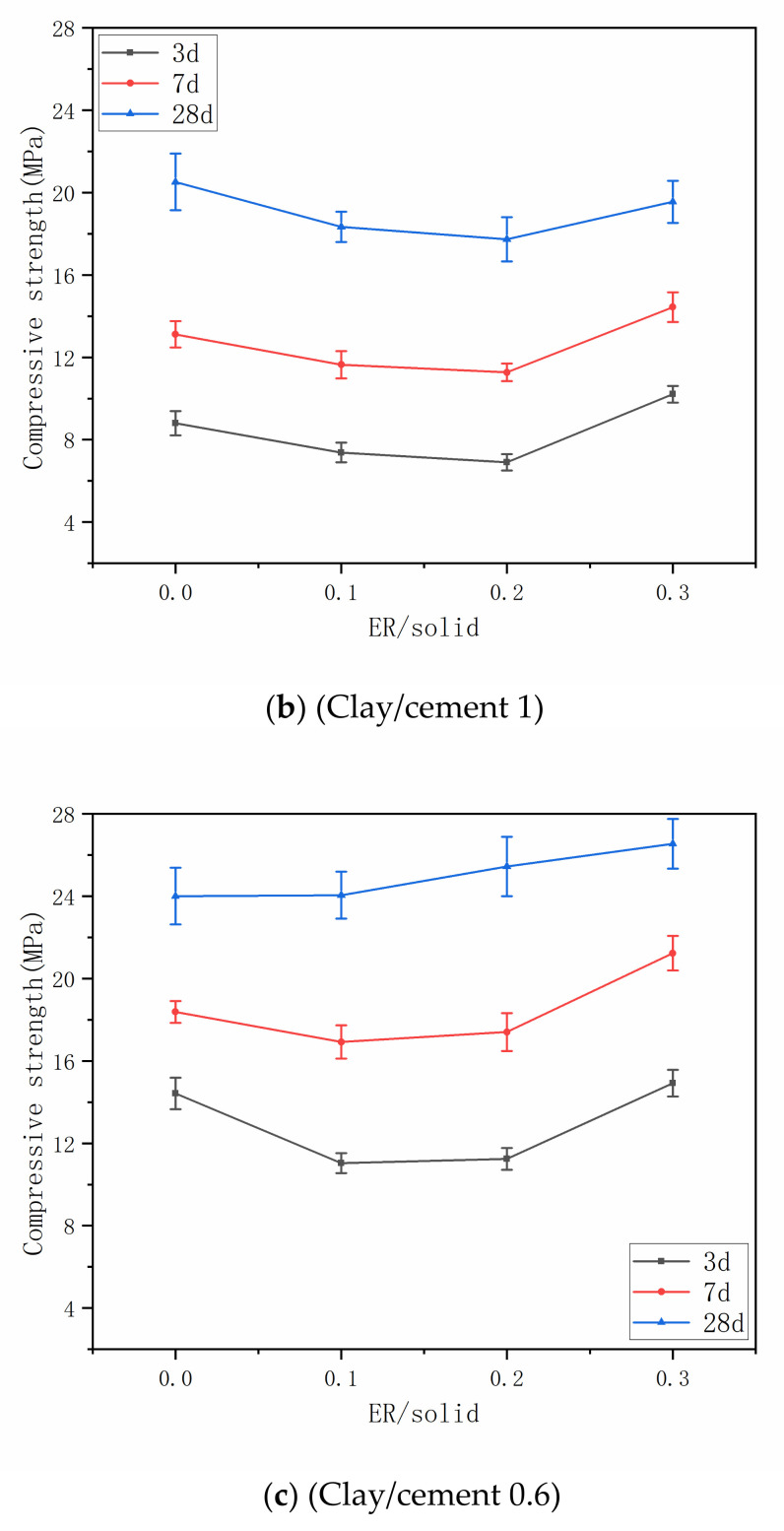
Compressive strength of the grouts with different ER/solid and clay/cement. (**a**) Clay/cement 1.4; (**b**) Clay/cement 1; (**c**) Clay/cement 0.6.

The compressive strength was measured. As shown in Figure 6, the compressive strength of each stone body increases with the increase of curing age, and the addition of resin improves the development speed of compressive strength. In addition, as the epoxy resin increases, the compressive strength initially decreases and then tends to increase. The above results show that the epoxy resin has a positive effect on the compressive strength. When the resin addition amount is low, it is diluted by water in the system, and the strength of resin itself is reduced. In addition, the bond between cement hydration products and clay particles is relatively weak, and the distribution of polymer film is uneven, resulting in the reduction of stone strength. When the amount of resin is enough, the surface of cement hydration products and clay particles will be enriched in large quantities of resin film [32]. This enrichment phenomenon will significantly improve the bond between cement hydration products and clay particles, and improve the structure of the stone body, make it more compact, so as to improve the compressive strength.

**Figure 7:**
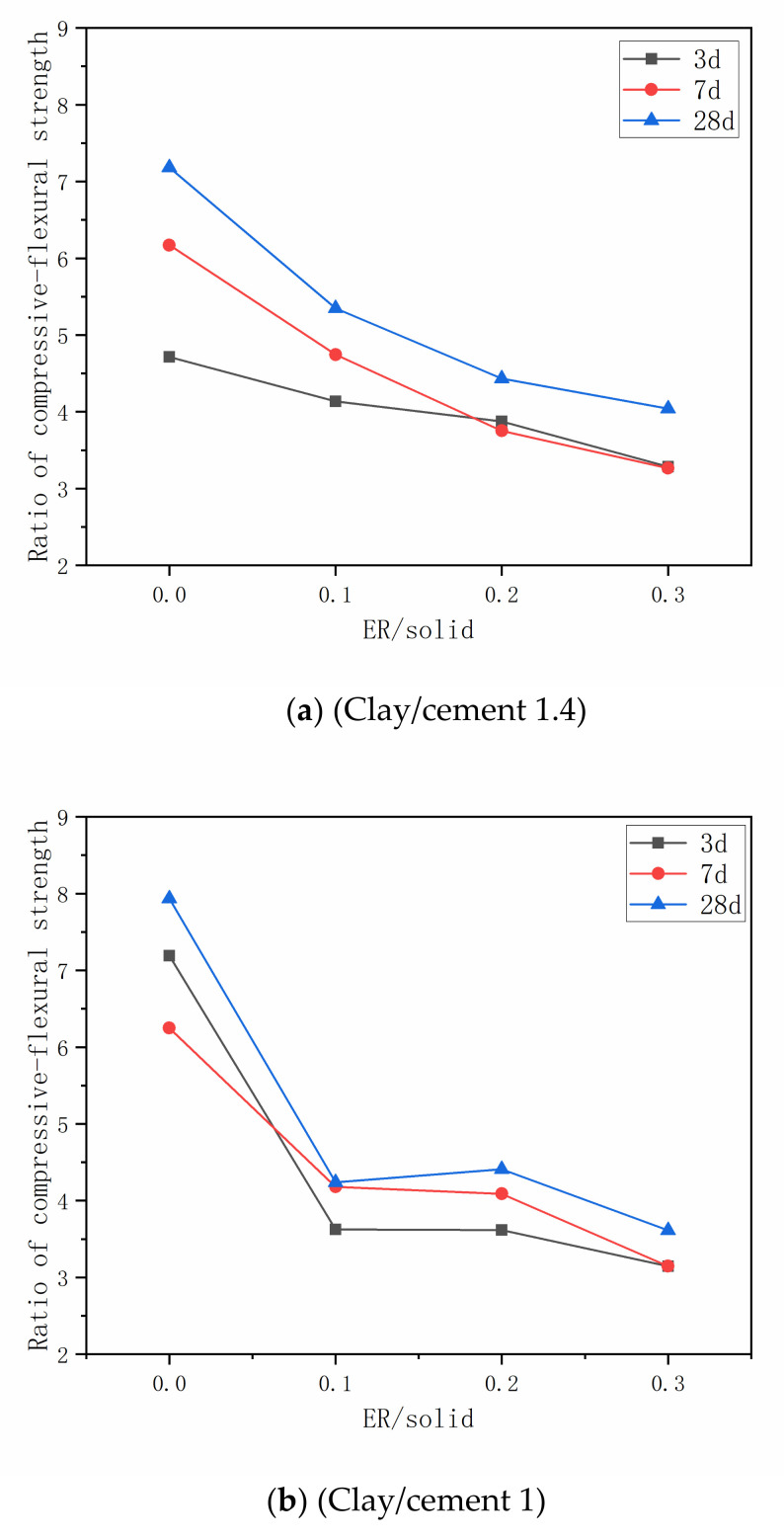

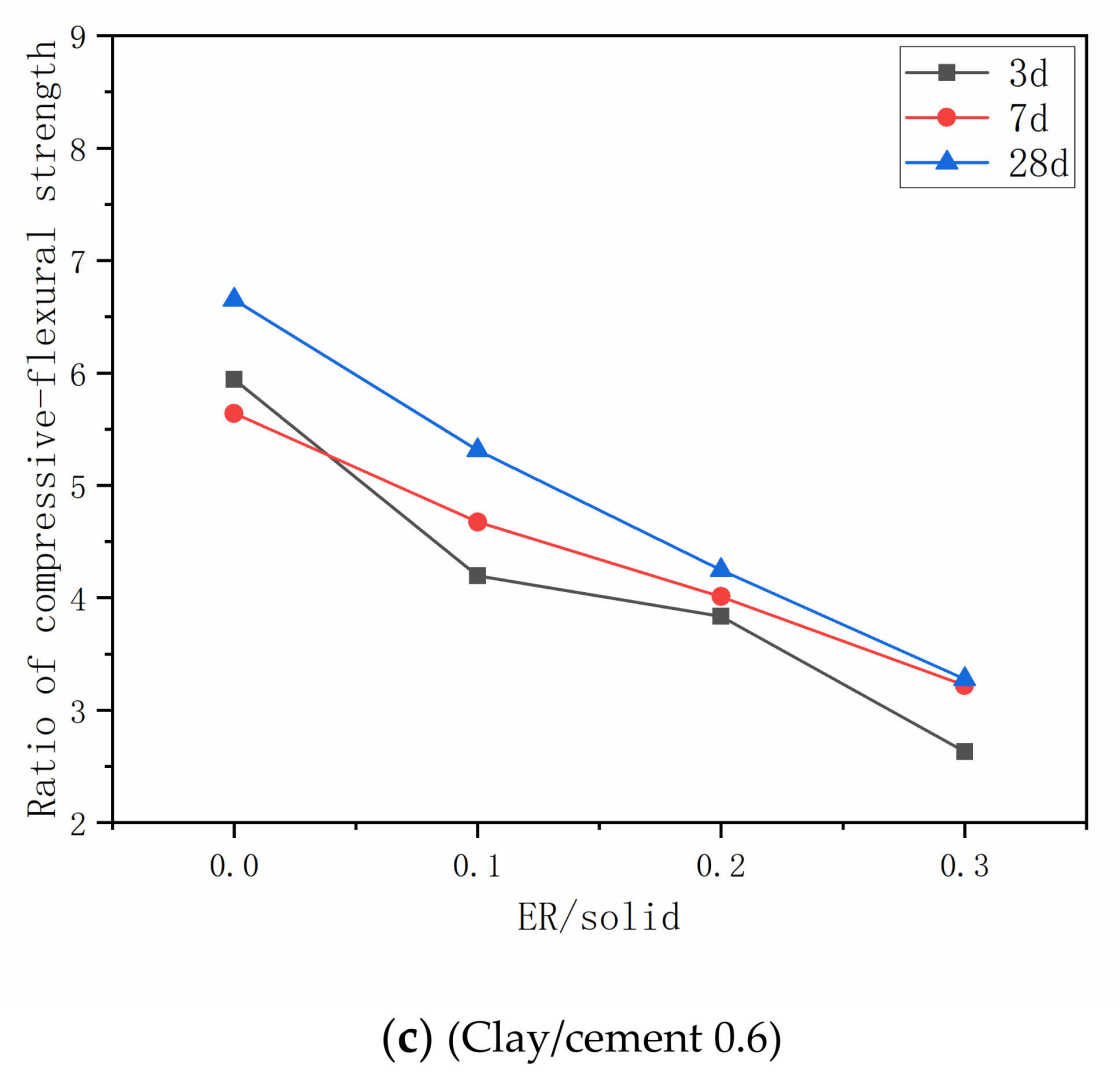
Development of the ratio of compressive-flexural strength. (**a**) Clay/cement 1.4; (**b**) Clay/cement 1; (**c**) Clay/cement 0.6.

Ratio of the compressive strength to the flexural strength of the material is one of the methods to characterize the toughness of cement-based materials. Figure 7 shows the development of the compression-flexural strength ratio of the grout with different proportion ratios. It can be seen that the compression-flexural strength ratio of the grout decreases significantly with the increase of the ER-solid ratio, which shows the brittleness of the stone body is decreased. When the resin particles disperse in the cement-clay slurry, polymer film is formed gradually around the hydration products and clay particles in the process of curing reaction, this kind of film fills in the pores and microfractures of the hardened cement-clay slurry and connect with each other, which improves the toughness of the grout [43].

**Figure 8:**
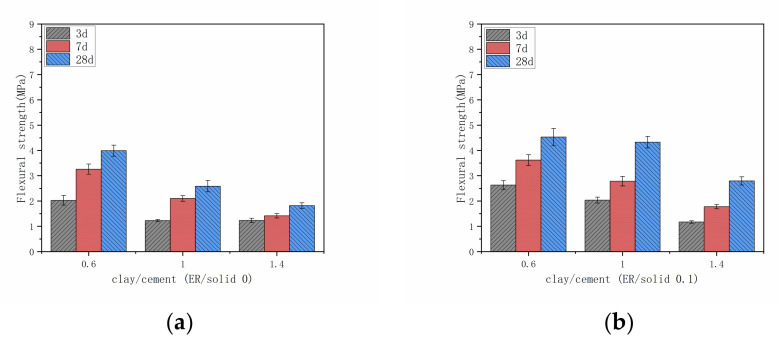

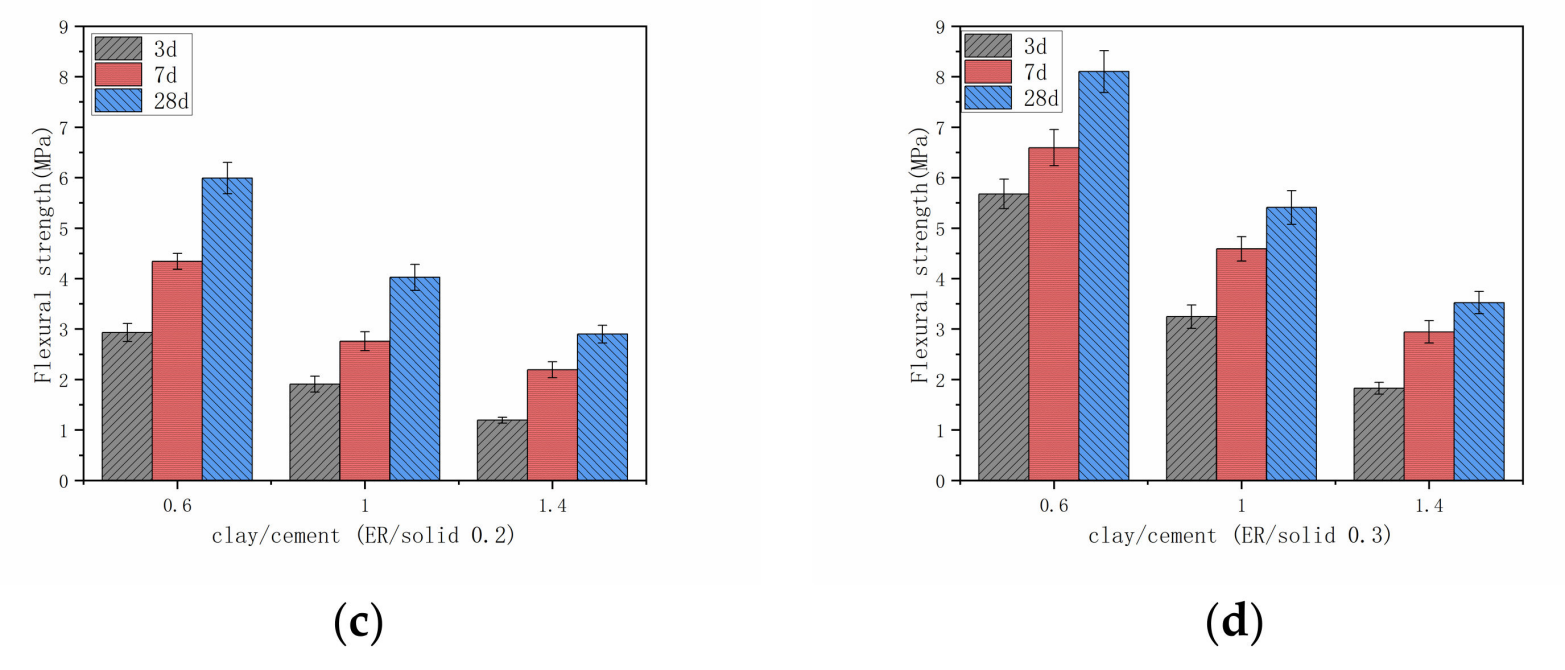
Flexural strength of grout with different admixtures of clay and cement. (**a**) ER/solid 0; (**b**) ER/solid 0.1; (**c**) ER/solid 0.2; (**d**) ER/solid 0.3.

**Figure 9:**
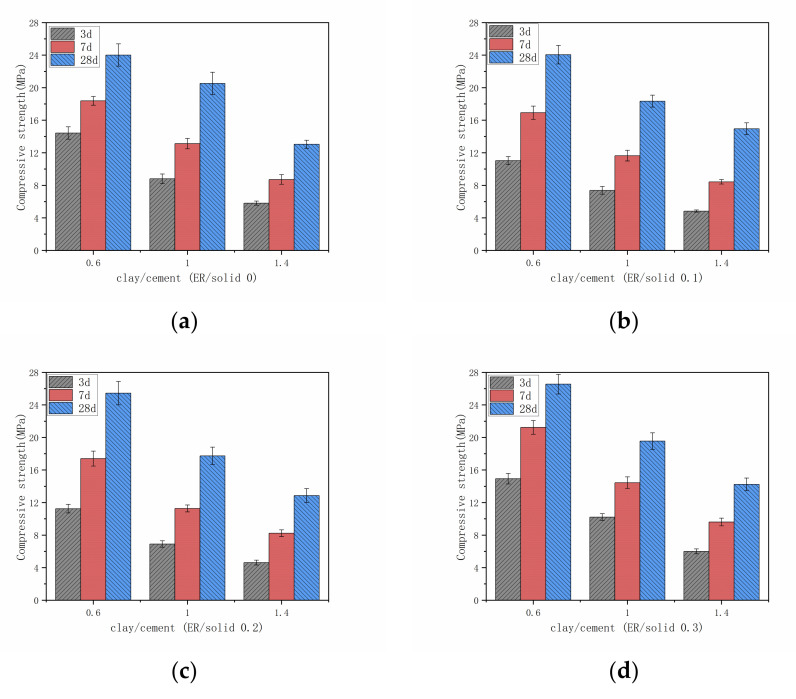
Compressive strength of grout with different admixtures of clay and cement. (**a**) ER/solid 0; (**b**) ER/solid 0.1; (**c**) ER/solid 0.2; (**d**) ER/solid 0.3.

According to Figure 8 and Figure 9, the flexural strength and compressive strength of the grout stone body are related to the content of clay and cement in the grout mix. From the results, the addition of cement improves the strengths of all mixes, which agrees with the test results of previous studies [35]. Due to the pozzolanic reactions, the addition of cement is very effective in the strength development of the grout. The pozzolanic reactions consume a large quantity of water, which would promote clay particles to lose their electrostatic attraction [44,45]. The clay in the grout mix is dispersed as flaky particles, the edges of the particles have a positive charge, and the surface is negatively charged. The edges and surface will attract each other to form a scaffold structure. The surface of the cement particles has a positive charge, which will attract clay particles and damage parts of the scaffold structure to form a clay cement ball with cement particle in the center. In these clay cement balls, cement hydration starts at the surface of the cement particles and spreads outward, filling the voids between the clay particles to build strength [46]. When the amount of cement is small, the scaffold structure cannot be completely destroyed in the grout system, so the scaffold structure and clay cement balls coexist. With the decrease of clay and the increase of cement, the scaffold structure of clay is destroyed. The cement hydrate gradually formed and connected into a skeleton. The clay particles and epoxy resin fill the pores and the strength is developed.

### 3.4. Microstructure Analysis

#### 3.4.1. SEM Analyses

**Figure 10:**
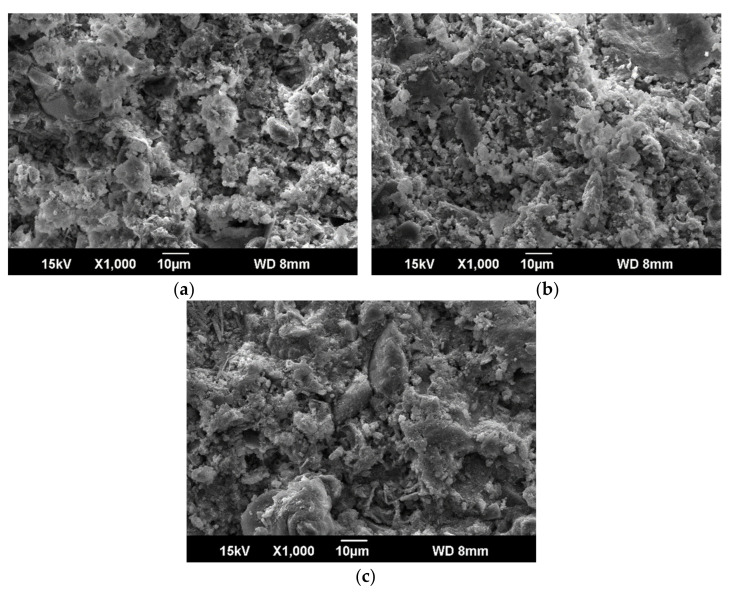
SEM (Scanning Electron Microscopy) of e-1 grout with different curing age. (**a**) Preserved for 3 days; (**b**) preserved for 7 days, and (**c**) preserved for 28 days.

The microstructure of the grout with different curing time and different dosage of ER was analyzed with scanning electron microscopy (SEM). Figure 10 shows the SEM images of grout e-1 cured for 3 days, 7 days, and 28 days. There are only clay, cement, water, and superplasticizer in e-1 grout, of which clay/cement is 1.4. With the increase of curing age, the interior of the stone body tends to be compact. In the grout, the hydration reaction of cement happened under the surrounding of clay completely, which is a kind of active substance [45]. With the hydration products of cement produced, part of hydration products continue to harden and form skeleton, others react with active clay particles, mainly for the ion exchange reaction and coagulation. Complex reaction of cement hydration occurs step by step in the system:

2 (3CaO·SiO_2_) + 6H_2_O = 3CaO·2SiO_2_·3H_2_O + 3Ca (OH)_2_,

2 (2CaO·SiO_2_) + 4H_2_O = 3CaO·2SiO_2_·3H_2_O + Ca (OH)_2_,

3CaO·Al_2_O_3_ + 6H_2_O = 3CaO·Al_2_O_3_·6H_2_O.

With the hydration reaction, when the newly added Ca^2+^ exceeds to a certain amount, it reacts with SiO_2_ and Al_2_O_3_ in the clay and gradually generates stable crystalline compounds. Because the specific surface area of the gel particles generated by cement hydration is much larger than that of cement particles, they have great surface energy and strong adsorption activity. They can be further combined with large soil aggregates, and seal the gap between the soil aggregates to form an overall connection [46].

SiO_2_ + Ca (OH)_2_ + nH_2_O = CaO·SiO_2_· (n + 1)H_2_O,

Al_2_O_3_ + Ca (OH)_2_ + nH_2_O = CaO·Al_2_O_3_· (n + 1)H_2_O.

**Figure 11:**
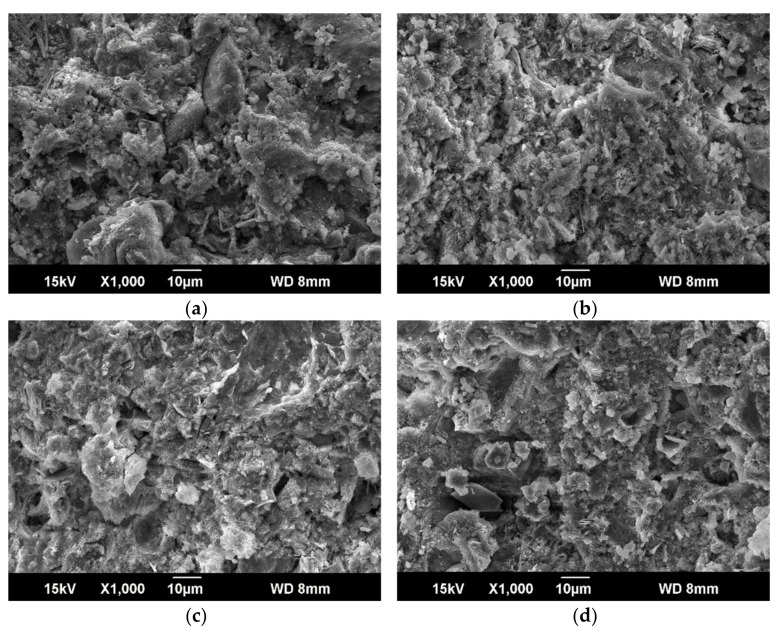
SEM of grout with different amount of ER. (**a**) e-1; (**b**) e-2; (**c**) e-3; (**d**) e-4.

Figure 11 shows the micrograph of the grout with different ER dosage. As shown in Figure 11, continuous polymer film structure appeared in the stone body after the addition of water-based epoxy resin, which changed the dispersion state of clay particles and hydration products, and increased the ductility inside the stone body. The flexural strength of the stone body increased with the increment of epoxy resin was verified. With the process of hydration, cement gel formed gradually, and resin particles began to partially deposit on the surface of cement gel, cement particles, and clay particles. When the cement gel structure further developed, resin particles gradually deposited into the pores. Hydration and water evaporation result in a decrease in free water, resin particles gradually packed tightly, at the same time, curing agent in the remaining water diffused into the epoxy resin particles and the crosslinking film started to form [47]. This crosslink structure not only fills the voids in the mixture, but also effectively binds cement hydration gel cement particles and clay particles together.

#### 3.4.2. XRD Analyses

**Figure 12:**
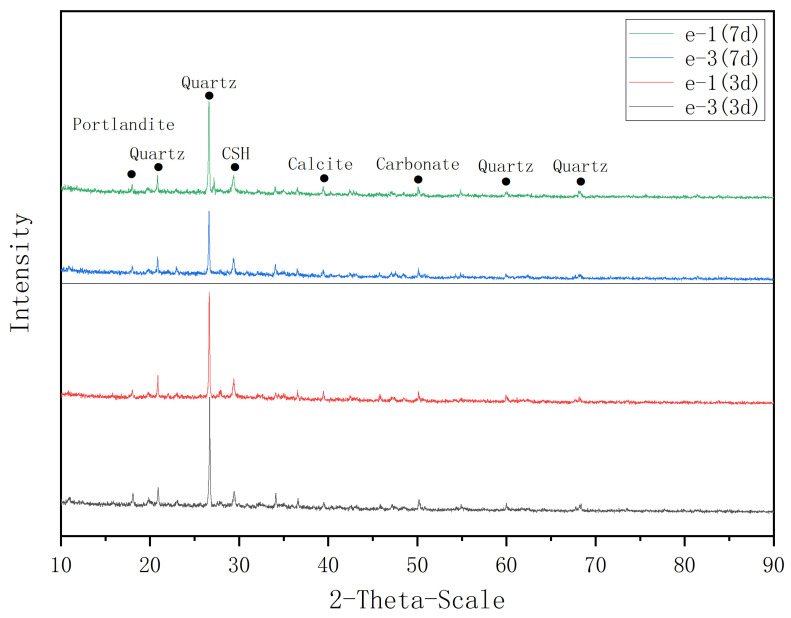
XRD spectra of grout e-1 and e-3 curing for 3 days and 7 days.

In order to analyze the early hydration process in the grout, XRD analysis was performed. The results of the XRD analyses conducted on the grout with different ER dosage (e-1 and e-3) and different curing time (3 days and 7 days) was shown in Figure 12. It can be observed that the characteristic peak of CSH increased slowly after the addition of epoxy resin, indicating that epoxy resin delayed the early hydration process of cement. CSH gel is the main contributor to the strength of cement [48]. The cohesion of CSH gel comes from the bonding force between colloidal particles, including the large van der Waals force between colloidal particles, the chemical and hydrogen bonding forces between water and Ca^2+^, OH^-^, and SiO_4_^4-^ in the gel. Low dosage of epoxy resin causes the decrease of compressive strength can be attributed to organic material destroyed the original ionic bonds between inorganic systems. However, the ionic and hydrogen bond, van der Waals forces and the interaction crosslink forces between the polymer and the inorganic system are not enough to compensate for the lost bond forces at a certain dosage, resulting in a decrease in compressive strength [43]. With the progress of hydration, the amount of Ca (OH)_2_, CSH, and ettringite increased continuously, forming contact points, which connected the particles into a network. With the increase of the number of contact points, the network structure continued to strengthen, making the structure denser.

## 4. Conclusions

In this paper, a new type of synchronous grouting material with high performance was developed. The results of the laboratory experiments led to conclusions about the workability, mechanical properties, and microstructure of the grout. Main conclusions can be summarized as follows:

(1) The addition of waterborne epoxy resin can reduce the density of the grout, improve the fluidity of the grout, the grout is stable with high stone rate.
(2) The mechanical properties of grout increased with the increase of epoxy resin content, but decreased with the increase of clay content.
(3) Microstructure test showed that the proper amount of epoxy resin mixed into cement clay slurry can combine well with hydration products and form a network structure, which can not only improve the hydration products, but also connect clay particles and hydration products, absorb energy of external load, and delay or restrain the development of the cracks, so as to enhance the toughness of the grout.

## Figures and Tables

**Table 1:** Chemical composition of the cement.

**Constituents**	**CaO**	**Al_2_O_3_**	**SiO_2_**	**Fe_2_O_3_**	**MgO**	**SO_3_**	**TiO_2_**	**L.O.I.**
%	68.50	1.28	18.40	2.73	5.48	2.45	1.04	0.12

**Table 2:** Chemical composition of the clay.

**Constituents**	**CaO**	**Al_2_O_3_**	**SiO_2_**	**Fe_2_O_3_**	**MgO**	**SO_3_**	**K_2_O**	**L.O.I.**
%	1.50	17.80	68.40	6.73	2.96	0.45	2.04	0.12

**Table 3:** Mineral components of the clay.

Constituents	Illite/Smectite Mixed-Layer	Illite	Kaolinite	Ratio of Mixed-Layer
%	56.00	19.00	25.00	50.00

**Table 4:** Composition of the tested grouts.

Designation	Proportion (Solid: Clay/Cement)	Proportion (Epoxy Resin (ER)/Solid)
e-1	1.4	0
e-2	1.4	0.1
e-3	1.4	0.2
e-4	1.4	0.3
e-5	1.0	0
e-6	1.0	0.1
e-7	1.0	0.2
e-8	1.0	0.3
e-9	0.6	0
e-10	0.6	0.1
e-11	0.6	0.2
e-12	0.6	0.3

**Table 5:** Test results of setting time.

**Designation**	**e-1**	**e-2**	**e-3**	**e-4**	**e-5**	**e-6**	**e-7**	**e-8**	**e-9**	**e-10**	**e-11**	**e-12**
Initial setting time (min)	325	332	334	352	293	326	330	347	268	279	294	322
Final setting time (min)	560	539	541	547	471	509	527	532	451	468	496	514

**Table 6:** Test results of bleeding rate and stone rate.

Designation	e-1	e-2	e-3	e-4	e-5	e-6	e-7	e-8	e-9	e-10	e-11	e-12
Bleeding rate (%)	0	0.5	0	0	0	1	0	0	0.5	1	1	0
Stone rate (%)	99.4	99.1	99.7	99.3	99.4	99.2	99.4	99.3	99.4	98.6	98.6	97.8

## Data Availability

The data presented in this study are available in insert article.
